# Analysis of Endoscopic Evaluation Reliability for Ulcerative Colitis in Histological Remission

**DOI:** 10.3390/healthcare9111405

**Published:** 2021-10-20

**Authors:** Mimari Kanazawa, Keiichi Tominaga, Akira Yamamiya, Takanao Tanaka, Shoko Watanabe, Takeshi Sugaya, Keiichiro Abe, Akira Kanamori, Takahiro Arisaka, Koki Hoshi, Makoto Iijima, Kenichi Goda, Yasuo Haruyama, Atsushi Irisawa

**Affiliations:** 1 Department of Gastroenterology, Dokkyo Medical University, Tochigi 321-0293, Japan; mimari77@dokkyomed.ac.jp (M.K.); akira-y@dokkyomed.ac.jp (A.Y.); tana1986@dokkyomed.ac.jp (T.T.); shoko-t@dokkyomed.ac.jp (S.W.); t-sugaya@dokkyomed.ac.jp (T.S.); abe9841@dokkyomed.ac.jp (K.A.); k-akira@dokkyomed.ac.jp (A.K.); aritaka@dokkyomed.ac.jp (T.A.); hoshi@dokkyomed.ac.jp (K.H.); mkiijima@dokkyomed.ac.jp (M.I.); goda@dokkyomed.ac.jp (K.G.); irisawa@dokkyomed.ac.jp (A.I.); 2 Integrated Research Faculty for Advanced Medical Science, Dokkyo Medical University, Tochigi 321-0293, Japan; yasuo-h@dokkyomed.ac.jp

**Keywords:** endoscopic remission, histological remission, interobserver reliability, Mayo endoscopic subscore, ulcerative colitis

## Abstract

The Mayo endoscopic subscore (MES) is a major endoscopic scoring system used to assign a status of mucosal inflammation and disease activity to patients with ulcerative colitis (UC). Using interobserver reliability (IOR), this study clarified the difficulties for endoscopic observers imposed by MES parameters used for the endoscopic evaluation of UC in histological remission. First, 42 endoscopists of four observer groups examined each MES parameter, which were evaluated from endoscopically obtained images of 100 cases as Grade 0 or 1 of the Nancy histological index of histopathological inflammation. Then, IOR was assessed using multiple κ statistics for each finding of MES. The results showed that IOR among all the observers was slight or fair for all the parameters, indicating a low IOR. The experts of the UC practice group had “moderate” or higher IOR for seven of the nine parameters, whereas “slight” or “fair” results were found for all parameters by the trainee group. The IOR for each MES parameter was calculated separately for the observer groups. All the groups showed “slight” or “fair” for “Erythema” and “Decreased vascular pattern”. Large differences between the endoscopists were found in the IOR for the MES parameters in UC in histological remission. Even among UC practice experts, the IOR was low for “Erythema” and “Decreased vascular pattern”.

## 1. Introduction

During the management of ulcerative colitis (UC), life events such as school attendance, employment, marriage, pregnancy, and baby delivery are possible when the long-term maintenance of remission is achieved [1,2]. Endoscopic remission (ER) is important as a short-term therapeutic goal leading to the achievement of long-term, therapeutic goals. The importance of the Treat to Target strategy has been proposed, through which treatment is organized to achieve ER [3,4].

Endoscopic evaluation is crucially important for UC management and treatment [1]. Several scores have been used to characterize and calculate the endoscopic findings for UC [5,6,7]. Among them, the Mayo endoscopic subscore (MES) presented by Schroeder et al. in 1987 [8] is a major endoscopic score system for evaluating the status of mucosal inflammation and disease activity. It remains the most commonly used endoscopic evaluation scale [9,10,11]. As an index of endoscopic activity based only on endoscopic mucosal findings, the MES system is used frequently. Nevertheless, it is a subjective evaluation; a different evaluation of the same endoscopic image by observers is common. The objectivity of evaluation has been investigated using various methods. The diagnostic criteria used for endoscopy are more reliable. Their use is reported more commonly for cases in which interobserver reliability (IOR) among endoscopists is higher. Travis SP et al. reported aspects of the ulcerative colitis endoscopic index of severity (UCEIS); its components show satisfactory intra- and inter-investigator reliability [12]. A systematic review indicated that the sigmoidoscopic component of MES and UCEIS presented the most promise as reliable evaluative instruments of endoscopic disease activity [13]. As described above, different studies have positively evaluated the validity and reliability of the endoscopic criteria that are commonly used for UC today. However, in several cases, ER was observed without full achievement of histological remission [14,15,16]. Some reports describe that the histological activity and endoscopic activity are correlated [17,18] but endoscopic findings with a low activity must be assessed for patients who have achieved histological remission. For this reason, a high interobserver reliability (IOR) is necessary for endoscopic findings to be assessed as such. Nevertheless, no report described the evaluation of the IOR of each MES parameter in patients who had achieved histological remission.

This study was designed to evaluate the IOR for each finding among the endoscopists for each MES item used for the endoscopic evaluation of UC cases that achieved histological remission. Then, difficulties with the MES items were clarified.

## 2. Materials and Methods

### 2.1. Study Design and Ethics

This study was approved by the ethics committee of Dokkyo Medical University Hospital (approval no. R-36-7J), conducted in accordance with the ethical principles stipulated in the Declaration of Helsinki, and registered with the University Hospital Medical Network Clinical Trials Registry (R000051904). Regarding the use of endoscopic photographs of patients, we provided a means to opt out instead of omitting informed consent, which was a way to guarantee an opportunity for research participants to notify and publish research information from our website.

### 2.2. Collection of Endoscopic Images and Histological Evaluation

**Figure 1:**
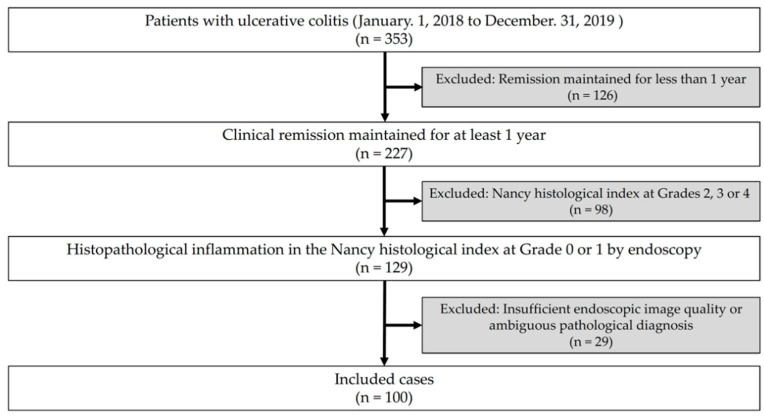
Flow diagram of this study.

From the medical chart database, among 353 patients treated for UC at the Department of Gastroenterology of Dokkyo Medical University Hospital from 1 January 2018 to 31 December 2019, data of patients that maintained clinical remission for at least 1 year (clinical remission was defined as a partial Mayo score of 3 points or lower [8], excluding 126 cases for which remission was maintained for less than 1 year), and of patients who were judged as Grade 0 or 1 of the Nancy histological index [19] of histopathological inflammation from periodic endoscopy (excluding 98 cases with Grade 2, 3 or 4 of Nancy histological index), were extracted to collect colonoscopic images at the time of pathological diagnosis. Pathological examinations were performed by two pathologists who specialized in pathology of gastrointestinal diseases. The degree of inflammation was assessed using the Nancy histological index based on the agreement of those two pathologists. From those cases, 29 cases judged by the principal investigator as having an endoscopic poor quality image and ambiguous pathological diagnosis were excluded. Therefore, 100 patients were selected for this study (Figure 1). For those 100 investigated cases, the clearest image was selected by MK for each case from endoscopic images of the site where the histopathological biopsy was conducted. The cases were selected by MK; 100 images were presented to the observer endoscopists without patient information. Therefore, the observer endoscopists were unable to obtain information related to histologic activity to exclude bias from the endoscopic evaluation. Additionally, MK was not included among the observers in this study.

### 2.3. Observers for IOR Evaluation

The images described above were shared with 42 endoscopists from our department, including trainers and trainees. They were evaluated to assess their MES classification. These 42 endoscopists were classified into the following four groups based on the number of cases experienced, years of experience and expertise: group A endoscopists examined at least 200 IBD patients per year and were certified as Board Certified Fellows of the Japan Gastroenterological Endoscopy Society (5 persons who were experts); group B endoscopists were not specialized in IBD treatment but were certified as Board Certified Fellows of the Japan Gastroenterological Endoscopy Society (14 persons); group C endoscopists had at least six years of clinical experience as gastroenterologists but were not certified as Board Certified Fellows of the Japan Gastroenterological Endoscopy Society (16 persons); and group D endoscopists were trainees with fewer than six years of clinical experience as a gastroenterologist (7 persons who were trainees).

### 2.4. Method of Presenting Endoscopic Findings

**Figure 2:**
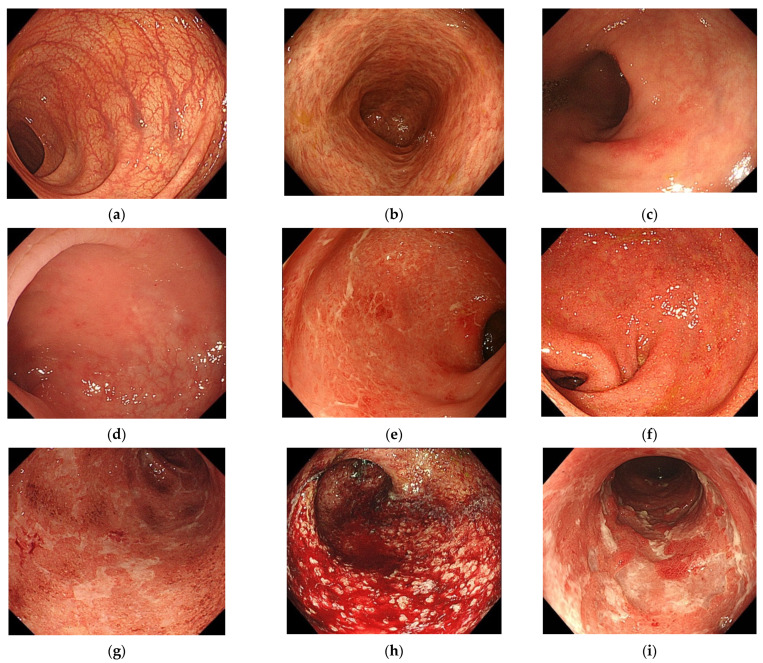
Endoscopic findings in Mayo endoscopic subscore: (**a**) normal, (**b**) inactive disease, (**c**) erythema, (**d**) decreased vascular pattern, (**e**) marked erythema, (**f**) absent vascular pattern, (**g**) erosions, (**h**) spontaneous bleeding, and (**i**) ulceration.

Endoscopic findings led to assignment of an MES [8,20] as MES 0 (normal, inactive disease), MES 1 (erythema, decreased vascular pattern, mild friability), MES 2 (marked erythema, absent vascular pattern, friability, erosions), or MES 3 (spontaneous bleeding, ulceration). Mild friability and normal friability among these findings were necessarily evaluated in real-time during endoscopy. They were excluded from the selected parameters because evaluating them in one presented image was expected to be too difficult. One hundred endoscopic images selected by MK were presented to observers to evaluate the presence or absence of endoscopic findings (nine selected parameters excluding mild friability and normal friability: normal (a), inactive disease (b), erythema (c), decreased vascular pattern (d), marked erythema (e), absent vascular pattern (f), erosions (g), spontaneous bleeding (h), and ulceration (i) (Figure 2)). The evaluator was not informed that this case had a Nancy histological index of 0 or 1.

For this study, IOR analysis was conducted for each endoscopic finding (multiple κ statistics).

### 2.5. Outcomes

For assessing the primary outcome of the present study, IOR was calculated among all observers for the MES parameters used for the endoscopic evaluation of UC. The secondary outcome was a comparison of findings obtained for the MES parameters among the four groups.

### 2.6. Statistical Analyses

Statistical analyses were performed using software (SPSS v.27.0; IBM SPSS Inc., Chicago, IL, USA). The Fleiss multirater kappa was used for IOR analysis [21]. Agreement was inferred from κ statistics in the standard fashion: 0.00, no agreement; 0.00–0.20, slight agreement; 0.21–0.40, fair agreement; 0.41–0.60, moderate agreement; 0.61–0.80, substantial agreement; and 0.81–1.00, almost perfect agreement [22]. Statistics calculated for this study were evaluated by a statistician using the blind test.

## 3. Results

### 3.1. IOR among All Observers for MES Parameters

The values of IOR calculated for all observers (42 persons) were calculated for the MES parameters (Table 1) as described below.

The interobserver κ coefficients for the respective endoscopic features of UC were 0.402 ± 0.003 in normal, 0.389 ± 0.003 for inactive disease, 0.235 ± 0.003 for erythema, 0.215 ± 0.003 for decreased vascular pattern, 0.351 ± 0.003 for marked erythema, 0.399 ± 0.003 for absent vascular pattern, 0.354 ± 0.003 for erosions, 0.1 ± 0.003 for spontaneous bleeding, and 0.212 ± 0.003 for ulceration. Only spontaneous bleeding was evaluated as “slight”; the other parameters were evaluated as “fair”.

### 3.2. Comparison of IORs among Observer Groups

The values of the IOR parameters consisting of MES were compared among the four observer groups (Table 2). The κ coefficients of the four observer groups differed. In Group A, the κ coefficient was “moderate” or higher for seven of the nine parameters. In Group B and Group C, the κ coefficients were “moderate” or higher for two of the nine and four of the nine parameters, respectively. In Group D, they were “slight” or “fair” in all the parameters.

### 3.3. IORs of MES Parameters by Observer Group

The IORs of MES parameters were calculated for the respective observer groups (Table 2). This investigation was conducted without Group D because all the parameters were evaluated as “slight” or “fair” in Group D.

For “Normal”, the κ coefficient was “moderate” in Groups A and C, whereas it was “fair” in Group B. For “Inactive disease”, the κ coefficient was “moderate” in Groups A, B, and C. The κ coefficient was found to have low values for “Erythema” and “Decreased vascular pattern”. They were “fair” or “slight” in all Groups. For “Marked erythema”, it was “moderate” only in Group A and was “fair” in Groups B and C. For “Absent vascular pattern”, it was “moderate” in Groups A and C, but it was “fair” in Group B. For “Erosion”, the κ coefficient moved from “substantial” to “moderate” in Groups A, B, and C. For “Spontaneous bleeding”, it was “almost perfect” in Group A, but the result was as low as “slight” in Groups B and C. For “Ulceration”, it was “moderate” in Group A, but the result was as low as “fair” or “slight” in Groups B and C.

## 4. Discussion

### 4.1. Meaning of MES

The lower gastrointestinal endoscopy for UC treatment is an important tool for making a diagnosis, elucidating clinical conditions, evaluating treatment, and for detecting and monitoring cancer. Endoscopic observations of inflammation in UC are scored using an objective indicator. In actual clinical situations, the Baron index [6,23] and Matts classification [24] are used. Actually, MES has been used more in recent, large-scale clinical studies [9,10,11].

### 4.2. Difficulties of Endoscopic Diagnosis and IOR in Image Diagnosis

Although the evaluation of the endoscopic findings using MES is important for the treatment selection and follow-up after treatment of UC, difficulty persists in the endoscopic diagnosis of UC: the interobserver agreement rate is unstable. Daperno et al. [25] analyzed the MES agreement rates reported by IBD experts and by IBD non-experts, and found poor results. The respective kappas of the IBD expert group and the IBD non-expert group were 0.53 and 0.71. One report also described that the perfect agreement rate of judgment as MES 0 or MES 1 was 68.2%, even among three endoscopists specializing in IBD [26].

A decrease in MES by at least 1 point is often regarded as an endoscopic improvement; MES 0 or MES 1 is often regarded as signifying an endoscopic remission [27,28]. However, it has been reported from recent studies that the relapse rate and the surgery rate are lower for MES 0 than for MES 1 [29,30,31]. Particularly, it was demonstrated that the remission maintenance rate differed in MES 1 by histological evaluation [15,32]. These problems might result from confusing and complicated parameters for endoscopic evaluation. Therefore, it is particularly important to improve the accuracy of judgment on endoscopic findings in the remission phase. Reportedly, endoscopic activity is correlated with histological activity [17,18], although the long-term studies of hospitalization rates and corticosteroid application rates have shown lower rates in histological remission than in endoscopic remission [16]. The period of remission maintenance is extended considerably in cases that have reached histological remission [33]. These findings suggest that histological remission can be a better indicator of remission maintenance than endoscopic remission. One reason for this might be the reliability of the endoscopic findings, i.e., IOR. Particularly, patients who have achieved histological remission often show endoscopic findings with low activity. This remission might lead to low IOR. In light of that possibility, we investigated the rate of agreement of endoscopic findings for UC patients in the histological remission phase among endoscopic observers. The results could indicate the reliability of evaluations made by endoscopists based on endoscopic data and images.

### 4.3. Significance of Study Results

The results of this study show the IOR of all the observers as “slight” for “Spontaneous bleeding” and “fair” for the other parameters, indicating somewhat lower results for the IOR because the IOR in Groups B, C, and D was lower than in Group A. Although Group B comprised endoscopists certified as Board Certified Fellows of the Japan Gastroenterological Endoscopy Society, pancreatobiliary work was a sub-specialty for most of them. They did not usually engage in IBD treatment, which might have affected the results. Group C members had no established sub-specialty, and therefore engaged in diverse treatments. The small number of cases they experienced might have affected their IOR. Particularly, Group D consisted of trainees with fewer than six years of clinical experience, resulting in the lower κ coefficient because of their relative lack of experience in endoscopy and their fewer cases experienced.

Regarding the item of “Spontaneous bleeding”, Group A comprising IBD specialists had a result of κ coefficient = 1, whereas the other three groups had a low IOR of ”slight”. The images presented for this study were endoscopic images showing histological remission. Therefore, the finding “Spontaneous bleeding” was not observed. However, observers other than the IBD experts tended to overestimate the findings and interpret them as showing “Spontaneous bleeding”.

By contrast, the parameters “Erythema” and “Decreased vascular pattern” elicited a low IOR as “fair” or “slight”, signifying a disagreement not only among IBD non-experts but among IBD experts. These parameters were similar in expression, expressed as “Erythema” and “Marked erythema”, as well as “Decreased vascular pattern” and “Absent vascular pattern”. Moreover, these findings are often observed simultaneously. It can be considered that this mode of expression led to a low IOR, eventually leading to results that were inappropriate as evaluation parameters. In fact, the overall IOR of MES overall was likely to be improved by changing these parameters to more objective ones for future use. Preparing several new expressions and findings, evaluating their resultant IOR, and choosing those which lead to better IOR as new evaluation parameters for newly modified MES is expected to be effective. Constructing more common and universal diagnostic parameters is desirable not only for IBD experts, but for all practitioners because all endoscopists might be involved in the endoscopic evaluation of IBD in actual clinical situations.

### 4.4. Limitations

There are some limitations to this study. First, the images used for evaluation in this study were not videos. They were one static image per case. Therefore, mild friability and friability, which served as real-time evaluation parameters during endoscopy, could not be included. Second, the study was a single-center study.

## 5. Conclusions

Large differences were found in the IOR of the MES parameters used by endoscopists for endoscopic evaluation of UC in the histological remission phase. Results indicate that IOR was low for the parameters “Erythema” and “Decreased vascular pattern”, even among experts at UC practice. The possibility exists that these MES parameters are inappropriate as evaluation parameters for endoscopic findings. The future analyses of the IOR in UCEIS can support development in this field.

## Figures and Tables

**Table 1:** Interobserver reliability of MES parameters for all 42 observers.

Feature	Interobserver (Multirater)	Evaluation
Normal		fair
κ	0.402 ± 0.003	
(95% CI)	(0.395–0.409)	
Inactive disease		fair
κ	0.389 ± 0.003	
(95% CI)	(0.382–0.395)	
Erythema		fair
κ	0.235 ± 0.003	
(95% CI)	(0.229–0.242)	
Decreased vascular pattern		fair
κ	0.215 ± 0.003	
(95% CI)	(0.208–0.222)	
Marked erythema		fair
κ	0.351 ± 0.003	
(95% CI)	(0.344–0.358)	
Absent vascular pattern		fair
κ	0.399 ± 0.003	
(95% CI)	(0.392–0.405)	
Erosions		fair
κ	0.354 ± 0.003	
(95% CI)	(0.348–0.361)	
Spontaneous bleeding		slight
κ	0.1 ± 0.003	
(95% CI)	(0.094–0.107)	
Ulceration		fair
κ	0.212 ± 0.003	
(95% CI)	(0.205–0.219)	

The Fleiss multirater kappa. κ statistics were interpreted in the standard fashion as follows: 0.00, no agreement; 0.00–0.20, slight agreement; 0.21–0.40, fair agreement; 0.41–0.60, moderate agreement; 0.61–0.80, substantial agreement; and 0.81–1.00, almost perfect agreement.

**Table 2:** Interobserver reliability of MES parameters for observer groups.

Feature	Endoscopists			
	Group A (*n* = 5)	Group B (*n* = 14)	Group C (*n* = 16)	Group D (*n* = 7)
Normal				
κ	0.457 ± 0.032	0.379 ± 0.01	0.492 ± 0.009	0.204 ± 0.022
% Agreement (95% CI)	55.2 (0.395–0.519)	49.8 (0.358–0.399)	61.2 (0.475–0.51)	43.2 (0.162–0.247)
Evaluation	moderate	fair	moderate	slight
Inactive disease				
κ	0.45 ± 0.032	0.486 ± 0.01	0.541 ± 0.009	0.068 ± 0.022
% Agreement (95% CI)	62.7 (0.388–0.512)	56.1 (0.466–0.507)	61.7 (0.523–0.559)	18.4 (0.025–0.111)
Evaluation	moderate	moderate	moderate	slight
Erythema				
κ	0.371 ± 0.032	0.197 ± 0.01	0.251 ± 0.009	0.209 ± 0.022
% Agreement (95% CI)	54.9 (0.309–0.433)	48.4 (0.176–0.217)	42.4 (0.233–0.269)	44.2 (0.166–0.252)
Evaluation	fair	slight	fair	slight
Decreased vascular pattern				
κ	0.067 ± 0.032	0.195 ± 0.01	0.312 ± 0.009	0.104 ± 0.022
% Agreement (95% CI)	36.6 (0.005–0.129)	56.3 (0.174–0.215)	57.2 (0.295–0.33)	37.4 (0.062–0.147)
Evaluation	slight	slight	fair	slight
Marked erythema				
κ	0.465 ± 0.032	0.281 ± 0.01	0.409 ± 0.009	0.272 ± 0.022
% Agreement (95% CI)	50 (0.403–0.527)	32.4 (0.26–0.302)	44.9 (0.391–0.427)	32.7 (0.229–0.315)
Evaluation	moderate	fair	fair	fair
Absent vascular pattern				
κ	0.458 ± 0.032	0.403 ± 0.01	0.47 ± 0.009	0.191 ± 0.022
% Agreement (95% CI)	56.6 (0.396–0.52)	53.2 (0.382–0.423)	57.6 (0.452–0.488)	29.7 (0.148–0.234)
Evaluation	moderate	fair	moderate	slight
Erosions				
κ	0.609 ± 0.032	0.464 ± 0.01	0.458 ± 0.009	0.147 ± 0.022
% Agreement (95% CI)	62.5 (0.547–0.671)	51.3 (0.444–0.485)	50.3 (0.44–0.476)	29.8 (0.105–0.19)
Evaluation	substantial	moderate	moderate	slight
Spontaneous bleeding				
κ	1	0.084 ± 0.01	0.116 ± 0.009	0.182 ± 0.022
% Agreement (95% CI)	100	10.8 (0.063–0.104)	12.4 (0.098–0.134)	19 (0.14–0.225)
Evaluation	almost perfect	slight	slight	slight
Ulceration				
κ	0.42 ± 0.032	0.215 ± 0.01	0.18 ± 0.009	0.169 ± 0.022
% Agreement (95% CI)	42.9 (0.358–0.482)	22.7 (0.194–0.235)	18.8 (0.162–0.197)	18.2 (0.126–0.212)
Evaluation	moderate	fair	slight	slight

The Fleiss multirater kappa. Κ statistics were interpreted in the standard fashion as follows: 0.00, no agreement; 0.00–0.20, slight agreement; 0.21–0.40, fair agreement; 0.41–0.60, moderate agreement; 0.61–0.80, substantial agreement; and 0.81–1.00, almost perfect agreement.

## Data Availability

No new data were created or analyzed in this study. Data sharing is not applicable to this article.

## References

[1] Kobayashi T., Siegmund B., Le Berre C., Wei S.C., Ferrante M., Shen B., Bernstein C.N., Danese S., Peyrin-Biroulet L., Hibi T. (2020). Ulcerative colitis. Nat. Rev. Dis. Primers.

[2] Ungaro R., Mehandru S., Allen P.B., Peyrin-Biroulet L., Colombel J.F. (2017). Ulcerative colitis. Lancet.

[3] Ungaro R., Colombel J.F., Lissoos T., Peyrin-Biroulet L. (2019). A Treat-to-Target Update in Ulcerative Colitis: A Systematic Review. Am. J. Gastroenterol..

[4] Colombel J.F., D’haens G., Lee W.J., Petersson J., Panaccione R. (2020). Outcomes and Strategies to Support a Treat-to-target Approach in Inflammatory Bowel Disease: A Systematic Review. J. Crohns Colitis.

[5] Rachmilewitz D. (1989). Coated mesalazine (5-aminosalicylic acid) versus sulphasalazine in the treatment of active ulcerative colitis: A randomized trial. BMJ.

[6] Hawthorne A.B., Logan R.F., Hawkey C.J., Foster P.N., Axon A.T., Swarbrick E.T., Scott B.B., Lennard-Jones J.E. (1992). Randomised controlled trial of azathioprine withdrawal in ulcerative colitis. BMJ.

[7] Travis S.P., Schnell D., Krzeski P., Abreu M.T., Altman D.G., Colombel J.F., Feagan B.G., Hanauer S.B., Lémann M., Lichtenstein G.R. (2012). Developing an instrument to assess the endoscopic severity of ulcerative colitis: The Ulcerative Colitis Endoscopic Index of Severity (UCEIS). Gut.

[8] Schroeder K.W., Tremaine W.J., Ilstrup D.M. (1987). Coated oral 5-aminosalicylic acid therapy for mildly to moderately active ulcerative colitis. A randomized study. N. Engl. J. Med..

[9] Sandborn W.J., Feagan B.G., Marano C., Zhang H., Strauss R., Johanns J., Adedokun O.J., Guzzo C., Colombel J.F., Reinisch W. (2014). Subcutaneous golimumab induces clinical response and remission in patients with moderate-to-severe ulcerative colitis. Gastroenterology.

[10] Sands B.E., Peyrin-Biroulet L., Loftus E.V., Danese S., Colombel J.F., Törüner M., Jonaitis L., Abhyankar B., Chen J., Rogers R. (2019). Vedolizumab versus Adalimumab for Moderate-to-Severe Ulcerative Colitis. N. Engl. J. Med..

[11] Feagan B.G., Danese S., Loftus E.V., Vermeire S., Schreiber S., Ritter T., Fogel R., Mehta R., Nijhawan S., Kempiński R. (2021). Filgotinib as induction and maintenance therapy for ulcerative colitis (SELECTION): A phase 2b/3 double-blind, randomised, placebo-controlled trial. Lancet.

[12] Travis S.P., Schnell D., Krzeski P., Abreu M.T., Altman D.G., Colombel J.F., Feagan B.G., Hanauer S.B., Lichtenstein G.R., Marteau P.R. (2013). Reliability and initial validation of the ulcerative colitis endoscopic index of severity. Gastroenterology.

[13] Samaan M.A., Mosli M.H., Sandborn W.J., Feagan B.G., D’Haens G.R., Dubcenco E., Baker K.A., Levesque B.G. (2014). A systematic review of the measurement of endoscopic healing in ulcerative colitis clinical trials: Recommendations and implications for future research. Inflamm. Bowel Dis..

[14] Bessissow T., Lemmens B., Ferrante M., Bisschops R., Van Steen K., Geboes K., Van Assche G., Vermeire S., Rutgeerts P., De Hertogh G. (2012). Prognostic value of serologic and histologic markers on clinical relapse in ulcerative colitis patients with mucosal healing. Am. J. Gastroenterol..

[15] Jangi S., Yoon H., Dulai P.S., Valasek M., Boland B.S., Jairath V., Feagan B.G., Sandborn W.J., Singh S. (2020). Predictors and outcomes of histological remission in ulcerative colitis treated to endoscopic healing. Aliment. Pharmacol. Ther..

[16] Bryant R.V., Burger D.C., Delo J., Walsh A.J., Thomas S., von Herbay A., Buchel O.C., White L., Brain O., Keshav S. (2016). Beyond endoscopic mucosal healing in UC: Histological remission better predicts corticosteroid use and hospitalisation over six years of follow-up. Gut.

[17] Rosenberg L., Nanda K.S., Zenlea T., Gifford A., Lawlor G.O., Falchuk K.R., Wolf J.L., Cheifetz A.S., Goldsmith J.D., Moss A.C. (2013). Histologic markers of inflammation in patients with ulcerative colitis in clinical remission. Clin. Gastroenterol. Hepatol..

[18] Nakazato Y., Naganuma M., Sugimoto S., Bessho R., Arai M., Kiyohara H., Ono K., Nanki K., Mutaguchi M., Mizuno S. (2017). Endocytoscopy is useful to assess histological healing in ulcerative colitis. Endoscopy.

[19] Marchal-Bressenot A., Salleron J., Boulagnon-Rombi C., Bastien C., Cahn V., Cadiot G., Diebold M.D., Danese S., Reinisch W., Schreiber S. (2017). Development and validation of the Nancy histological index for UC. Gut.

[20] Scherl E.J., Pruitt R., Gordon G.L., Lamet M., Shaw A., Huang S., Mareya S., Forbes W.P. (2009). Safety and efficacy of a new 3.3 g b.i.d. tablet formulation in patients with mild-to-moderately active ulcerative colitis: A multicenter, randomized, double-blind, placebo-controlled study. Am. J. Gastroenterol..

[21] Fleiss J.L. (1971). Measuring nominal scale agreement among many raters. Psychol. Bull..

[22] Landis J.R., Koch G.G. (1977). The measurement of observer agreement for categorical data. Biometrics.

[23] Baron J.H., Connell A.M., Lennard-Jones J.E. (1964). Variation between observers in describing mucosal appearances in proctocolitis. Br. Med. J..

[24] Matts S.G. (1961). The value of rectal biopsy in the diagnosis of ulcerative colitis. Q. J. Med..

[25] Daperno M., Comberlato M., Bossa F., Biancone L., Bonanomi A.G., Cassinotti A., Cosintino R., Lombardi G., Mangiarotti R., Papa A. (2014). Inter-observer agreement in endoscopic scoring systems: Preliminary report of an ongoing study from the Italian Group for Inflammatory Bowel Disease (IG-IBD). Dig. Liver Dis..

[26] Takahashi F., Tominaga K., Kanamori A., Takenaka K., Hoshino A., Sugaya T., Nakano M., Hiraishi H. (2016). Timing for dose-down of 5-ASA depends on mucosal status with ulcerative colitis. Scand. J. Gastroenterol..

[27] Barreiro-de Acosta M., Vallejo N., de la Iglesia D., Uribarri L., Bastón I., Ferreiro-Iglesias R., Lorenzo A., Domínguez-Muñoz J.E. (2016). Evaluation of the Risk of Relapse in Ulcerative Colitis According to the Degree of Mucosal Healing (Mayo 0 vs. 1): A Longitudinal Cohort Study. J. Crohns Colitis.

[28] Vuitton L., Peyrin-Biroulet L., Colombel J.F., Pariente B., Pineton de Chambrun G., Walsh A.J., Panes J., Travis S.P., Mary J.Y., Marteau P. (2017). Defining endoscopic response and remission in ulcerative colitis clinical trials: An international consensus. Aliment. Pharmacol. Ther..

[29] Yokoyama K., Kobayashi K., Mukae M., Sada M., Koizumi W. (2013). Clinical Study of the Relation between Mucosal Healing and Long-Term Outcomes in Ulcerative Colitis. Gastroenterol. Res. Pract..

[30] Mazzuoli S., Guglielmi F.W., Antonelli E., Salemme M., Bassotti G., Villanacci V. (2013). Definition and evaluation of mucosal healing in clinical practice. Dig. Liver Dis..

[31] Manginot C., Baumann C., Peyrin-Biroulet L. (2015). An endoscopic Mayo score of 0 is associated with a lower risk of colectomy than a score of 1 in ulcerative colitis. Gut.

[32] Kanazawa M., Takahashi F., Tominaga K., Abe K., Izawa N., Fukushi K., Nagashima K., Kanamori A., Takenaka K., Sugaya T. (2019). Relationship between endoscopic mucosal healing and histologic inflammation during remission maintenance phase in ulcerative colitis: A retrospective study. Endosc. Int. Open.

[33] Fujiya M., Saitoh Y., Nomura M., Maemoto A., Fujiya K., Watari J., Ashida T., Ayabe T., Obara T., Kohgo Y. (2002). Minute findings by magnifying colonoscopy are useful for the evaluation of ulcerative colitis. Gastrointest. Endosc..

